# Disaster Preparedness and Awareness among University Students: A Structural Equation Analysis

**DOI:** 10.3390/ijerph20054447

**Published:** 2023-03-02

**Authors:** Ronik Ketankumar Patel, Apurva Pamidimukkala, Sharareh Kermanshachi, Roya Etminani-Ghasrodashti

**Affiliations:** 1 Department of Civil Engineering, University of Texas at Arlington, Arlington, TX 76019, USA; 2 Center for Transportation Equity, Decisions and Dollars (CTEDD), University of Texas at Arlington, Arlington, TX 76019, USA

**Keywords:** student perception, disaster education, structural equation modelling, disaster curriculum, disaster preparedness

## Abstract

Students have long been among those most emotionally and physically affected by natural or manmade disasters, yet universities and colleges continue to lack effective disaster response and mitigation practices. This research identifies how students’ socio-demographics and disaster preparedness indicators (DPIs) impact their awareness of the dangers of disasters and their ability to survive and cope with the changes that disasters bring. A comprehensive survey was designed and distributed to university students to gain an in-depth understanding of their perceptions of disaster risk reduction factors. A total of 111 responses were received, and the impact of the socio-demographics and DPIs on the students’ disaster awareness and preparedness were evaluated by employing structural equation modeling. The results indicate that the university curriculum impacts the disaster awareness of students while the establishment of university emergency procedures impacts the disaster preparedness of students. The purpose of this research is to enable university stakeholders to identify the DPIs that are important to the students so that they can upgrade their programs and design effective DRR courses. It will also aid policymakers in redesigning effective emergency preparedness policies and procedures.

## 1. Introduction

A disaster is a hazardous event that disrupts the functioning of a society or community and causes human, material, environmental, and economic losses [1]. The four phases of disaster include mitigation, preparedness, response, and recovery [2,3]. The mitigation and preparedness phases occur before a disaster hits and facilitates realistic predictions of what it will affect. The response phases continue until immediately after the disaster, and the recovery phase extends until the regular operations and activities are again performed at a satisfactory level. Decisions that are made during the mitigating and preparedness phases highly impact the time and effectiveness of the response and recovery phases [4,5,6]. 

The number and severity of natural disasters has increased significantly in recent years [7,8]. There were only about 100 natural disasters reported annually worldwide during the 1980s, and this number has risen to over 300 since 2000. Disasters have impacted both developed and developing countries [9]. For example, the 2011 earthquake in Japan alone was responsible for the economic loss of USD 221.6 billion. In the United States, during the ten-year period of 2003 to 2013, natural disasters were responsible for damages amounting to USD 1.5 trillion; from 2016 to 2017, the losses were approximately USD 200 billion [10].

While most are familiar with the disruptions caused by disasters, many are not aware of the negative impacts that they have on students. Disasters affect students by disrupting campus activities, interrupting classes, and damaging school buildings [11]. In recent years, universities have begun to recognize the value of being prepared for disasters and their associated risks, and students have become more aware of disasters through personal experience, seminars, and the media. Disaster awareness denotes the extent of knowledge about disaster risks, and the factors that lead to disasters influence the actions that could be taken individually or collectively to address exposure and vulnerability to hazards, while disaster preparedness denotes the measures that are taken to prepare for/reduce the effects of disasters [6]. Despite the increase in awareness, however, many universities and schools still lack adequate planning, response, and mitigation strategies [12]. 

According to Tanner and Doberstein [13], students are the least-considered group of a community when plans are being made for emergency preparedness. Mulilis et al. [14] found similar results while evaluating tornado preparedness of students, non-students, tenants, and homeowners, and a study that was conducted in China [15] revealed that more than half of the students did not know basic survival skills, even though they were taught cardiopulmonary resuscitation (CPR). While administering CPR is an important skill, in the face of the increasing number of natural disasters that are occurring worldwide, it seems important for universities and schools to also provide education on other essential rescue skills. Doing so will increase students’ disaster preparedness and enable them to apply their skills during a disaster.

Awareness is important, but students must also be prepared for disasters by being taught the essential rescue skills that can significantly mitigate their effects [16,17,18]. These rescue techniques are a vital aspect of disaster education and should be taught by competent professionals [15]. Universities with nursing and/or medical schools have an advantage, as they have the instruments and professionals to develop and implement effective disaster training and courses [19]. All educators in institutions of higher learning can successfully develop and offer disaster risk reduction (DRR) courses, however, and their willingness and ingenuity will determine the quality of the courses [20]. Prepared students are more confident and more likely to use their knowledge of the physical and psychological barriers precipitated by disasters to assist local disaster management agencies [21]. 

Even though students are among the most vulnerable groups in the community to natural disasters, they are often overlooked [19,20]. University students are a rare set of individuals with a versatile worldview and exceptional adaptability [21]. They can learn emergency skills more rapidly and efficiently than the general population since they possess these characteristics. As training sessions might greatly lower the costs of the damages and students can be valuable resources for disaster response, prevention, and mitigation in general, it is strongly suggested to equip students with the right training and education [9,22].

Previous studies have demonstrated that implementing a university disaster preparedness course requires the difficult task of collaborating with local leaders [22]. Ideally, senior university administrators collaborate with local emergency management agencies to enhance the disaster preparedness of both the university and the community [23], while providing practical training and strengthening the relationship between them [22]. The financial capability of a university often plays an important role in the development and delivery of technical disaster education and/or towards the integration of DRR courses with other courses [24]. Amri et al. [20] found that most teachers believe that training would help them teach a DRR course more effectively; hence it follows that a collective effort by trained teachers could effectively lead to the successful application of disaster education. Recognizing and awarding effective and experienced distance education teachers may entice others to follow their footsteps [25].

The literature makes it clear that acquiring knowledge and skills helps students be prepared for disasters. Therefore, it is vital for universities to educate their students about how disasters can impact them and to equip them with the knowledge and skills to mitigate those impacts. Students can be valuable assets for the local community and management agencies during disaster recovery if they are trained and given the necessary tools. Thus, this study aims to fill the void in the literature on this subject by investigating university students’ knowledge and perceptions of disaster preparedness indicators (DPIs). We formulated three specific objectives for this study: (i) to identify the disaster preparedness indicators (DPIs), (ii) to identify the critical components that are associated with the DPIs using factor analysis, (iii) and to develop a structural equation model to evaluate the relationship between students’ socio-demographic characteristics and DPIs on disaster awareness and preparedness. This study’s findings will provide insights for faculty members, academic staff, and university policymakers and will enable them to make changes in existing policies and procedures, reform existing programs, enhance students’ disaster preparedness, and minimize the expensive and deadly impacts of disasters. 

### Disaster Preparedness Indicators

As the focus of the study is primarily to evaluate the relationship between students’ socio-demographic characteristics and DPIs on disaster awareness and preparedness, the DPIs were identified from the literature. Patel et al. [26] identified 24 DPIs, and the list of DPIs is presented in Table 1. 

## 2. Materials and Methods

**Figure 1:**
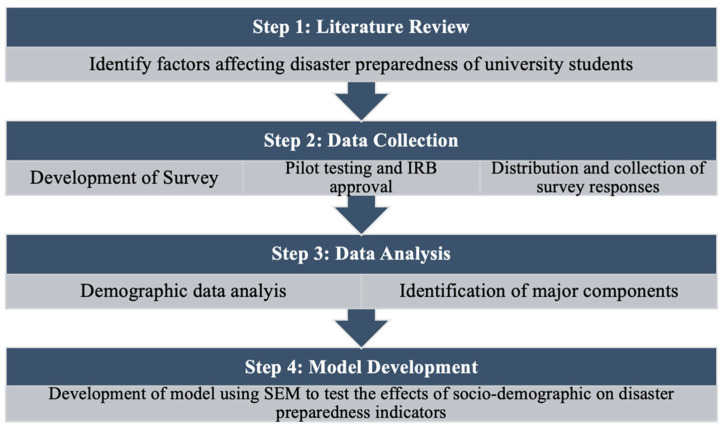
Research Method.

As presented in Figure 1, a four-step research methodology was adopted to fulfill the objectives of this study. In the first step, a comprehensive literature review was conducted to identify the factors that affect disaster preparedness of university students. In the second step, a comprehensive survey was developed from the identified literature, and was reviewed and approved by IRB, before distributing it to the participants. In the third step data analysis was performed on the socio-demographic data of the respondents, and major components were also identified. Finally, structural equation modeling (SEM) was adopted to develop a model to test the effects of sociodemographics on disaster preparedness indicators.

### 2.1. Data Collection 

After reviewing the existing literature on disaster risk reduction, a structured survey was developed. The survey was divided into six sections and consisted of 44 multiple choice and rating-scale questions. The rating-scale type questions employed a 7-point Likert scale. The survey was pilot tested by distributing it among a few Ph.D. students to ensure that the questions were clearly stated, after which the Institutional Review Board of the University of Texas at Arlington (UTA) reviewed and approved it. The survey was distributed to UTA students from different engineering majors via email, with a brief overview of the study. There was no remuneration for taking part in the study. 

The questions in the first section pertained to demographics, such as age, gender, ethnicity, level of education, residence (on-campus or off-campus), etc. The second segment had questions about students’ role in disasters to analyze their disaster experiences. The third section examined the students’ views of practical and theoretical disaster education and their willingness to register for such courses. University policies governing emergency preparedness were addressed in the fourth section, which also asked questions pertaining to first aid and the agencies that are responsible for students’ safety during an emergency. The fifth section had questions about the university’s emergency protocols and medical supplies, and the last section asked about disaster education implementation and obstacles to learning about disaster preparedness. The survey questions are provided in Supplementary File S1. 

The survey was distributed through the online platform, Qualtrics to over 300 regular and full-time UTA students older than 18 years from various engineering majors. After two rounds of reminder emails, a total of 111 complete responses were received. 

### 2.2. Statistical Tests 

#### 2.2.1. Dimensions Reduction: Principal Component Analysis

Principal component analysis (PCA) is a multivariate statistical approach that is used in the field of social science to transform multiple associated factors into a reduced set of factors known as principal components, which account for variability in the original dataset [27]. It projects the variables that should comprise the latent variables so a model can be developed and tested. 

#### 2.2.2. Structural Equation Modeling 

Structural equation modeling (SEM) has gained popularity recently for developing multivariate relationships and parsimonious models [28]. SEM not only validates hypothesized relationships but also provides new relationships between constructs and parameters based on modification indexes. SEM is also efficient in handling complex dependencies and provides flexibilities with sample numbers [29]. Some researchers have proposed a minimum number of samples (e.g., 100 or 200), while some researchers have 5–10 samples per parameter [28]. Therefore, based on the type of collected data, the SEM modeling technique was chosen as the best fit. 

### 2.3. Data Analysis

Most of the respondents were Asians below the age of 25 years who came from low-income households (median household income less than $60k). Approximately 65% of the respondents were graduate students; the other 35% were undergraduates. Less than half of the students’ (63%) had either no or very little experience with disasters. The primary types of disasters that students indicated having experienced were earthquakes (25%), thunderstorms (23%), and flooding (20%); hurricanes (5%) and tsunamis (3%) were the least experienced. Table 2 provides detailed descriptive statistics of the participants.

## 3. Results 

### 3.1. Willingness to Take DRR Course

The survey results indicated that 41% of the students were willing to take a DRR course and 74% had not taken a related course prior to attending college. This finding suggests that despite not having had prior education on the subject, most of the students polled were not interested in studying disaster education. An earlier study indicated that knowledge of how to prepare for a disaster ultimately leads to a higher level of disaster preparedness [23]; therefore, universities should establish awareness programs that impart the importance of disaster education.

### 3.2. Students’ Perception of Assisting Disaster Management Agencies

The results revealed that 60% of the students were confident that they could assist disaster management agencies during a disaster, which is in line with the outcome of the study [23] that showed that more than half (59%) of the students would volunteer to participate in the disaster recovery process. Students’ participation in community engagement programs and volunteer activities during a disaster can help both universities and local communities in the recovery process, but their effectiveness is dependent on their having some disaster-related training or experience.

### 3.3. Students’ Perception of First Aid 

Students were asked about their confidence level in providing basic first aid during an emergency, and approximately 39% of them indicated that they were not confident; one-third (29%) of them were neither confident nor unconfident. This finding suggests that many students are not familiar with basic first-aid practices and would not be of much help to themselves or others during a disaster. Universities (ideally, someone from a nursing school) should teach them practical rescue skills and basic medical training and provide opportunities for hands-on experience. Universities without a nursing school or similar department should partner with a local hospital or nursing school to develop a training course. 

The survey included questions pertaining to the availability of first aid kits at the students’ university, and the responses revealed that 26% of the undergraduate students and 65% of the graduate students felt that their university had a sufficient number of kits; 53% of the undergraduates and 24% of the graduate students were neutral. This finding obviously suggests a higher awareness of available medical supplies among graduate students. 

### 3.4. Students’ Perception of Who Is Responsible for Their Safety

The survey asked the students to rank the entities that are responsible for their safety during an emergency, and the results are shown in Table 3. The findings indicated that 76% (important or extremely important) of the students considered themselves to be responsible for their own safety during an emergency. In comparison, 57% believed that government agencies were obligated for their safety, followed by their university (49%), parents (45%), and friends (35%), respectively. 

### 3.5. Analysis of Students’ Perception of including DRR Education in the Curriculum

**Figure 2:**
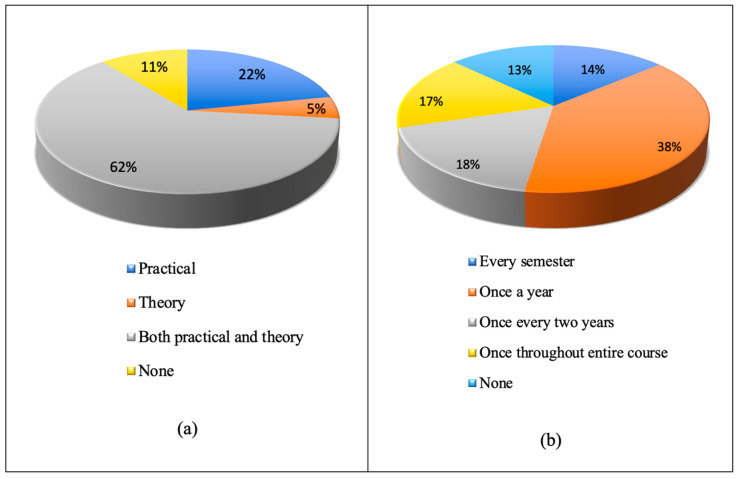
Pie charts showing students’ perception of (**a**) the form of DRR education that needs to be incorporated in a curriculum and (**b**) how often DRR education needs to be offered.

Figure 2a,b depict the form of education and frequency with which the students felt DRR classes should be offered, respectively. The results revealed that 62% of the students believed that both theoretical and practical DRR education are essential and 38% believed that it should be provided once every year. Accordingly, it is vital that DRR education be conducted either as a part of an existing course or by introducing a new class [30]. 

### 3.6. Analysis of Students’ Perception of Major Barriers to Learning about DRR

**Figure 3:**
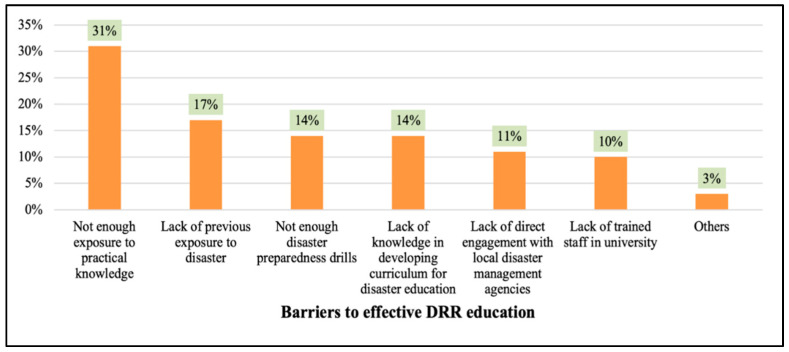
Analysis of students’ perception of barriers to effective DRR education.

The results of the survey revealed that 31% of the students believed that inadequate exposure to practical knowledge is a significant barrier to becoming well educated about DRR, 17% believed that lack of previous disaster experience is a major barrier, and 14% believed that too few disaster preparedness drills is a significant barrier (Figure 3).

### 3.7. Statistical Analysis 

#### 3.7.1. PCA

PCA with varimax (orthogonal) rotation was performed using SPSS AMOS V.28 software to extract latent factors from all DPIs that were discussed earlier (see Table 1). However, DPIs (#2, 3, 5, 6, 7, 8, 9, 10, 18, and 19) were excluded from the analysis due to poor factor loading. The analysis yielded six factors: government/university responsibility, emergency procedures, university curriculum, DRR adoption, disaster preparedness, and disaster awareness. The Kaiser Mayer Olkin (KMO) for the dataset was found to be greater than cutoff point 0.5, indicating that the data are suitable for performing factor analysis. Table 4 depicts the results from the factor analysis. 

#### 3.7.2. Conceptual Model

**Figure 4:**
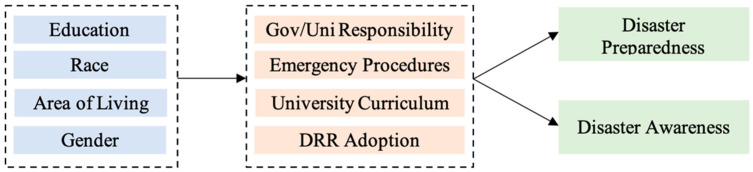
Expected Conceptual Model Before the Analysis.

Previous studies [31,32] focused on identifying the factors that affect the disaster preparedness of students based on their socio-demographics (gender, race, area of living, and education level). There were six key components (government/university responsibility, emergency procedures, university curriculum, DRR adoption, disaster preparedness, and disaster awareness) that were identified, based on the disaster preparedness indicators that were identified using factor analysis. We hypothesize that students’ socio-demographics also directly influence these key variables and indirectly affect their awareness of and preparedness for disasters. The conceptual framework that was adopted for this study is presented in Figure 4.

Structural equation modelling (SEM) was applied to evaluate the effects of different variables on disaster awareness and preparedness. In addition to exploring interdependencies among crucial components, SEM concurrently assesses the direct, indirect, and total effects. The expected conceptual model that was developed based on the factors extracted using factor analysis, is shown in Figure 4. 

**Figure 5:**
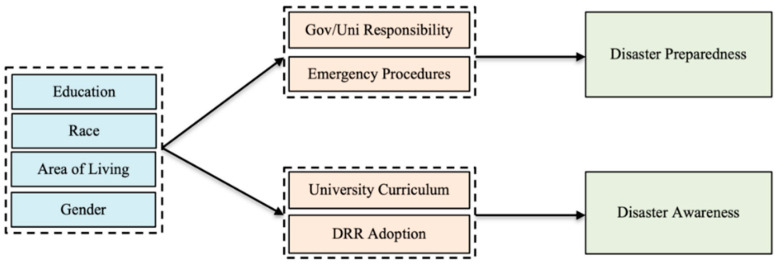
Verified Model After the Analysis.

These factors behave as observed variables and are considered endogenous variables in path analysis [33]. The students’ socioeconomic characteristics were targeted as exogenous variables since they influence the key variables but are not affected by other key variables. However, when the authors ran the analysis with the expected conceptual model, they did not find good results. As a result, authors had to remove some of the relationships to better fit the model. Figure 5 presents the verified model after the analysis.

### 3.8. SEM Modelling

#### 3.8.1. Mediating Effects of Key Variables

To examine the validity and reliability of the hypothesized model, three model fitness indices were tested to evaluate the difference between the observed and implied variance-covariance matrix. The value of chi-square divided by the degree of freedom (χ²/df = 1.9) indicates whether the data fits the model. If the value of χ²/df is less than 2, it suggests that the model is a good fit [34]. Secondly, the root mean square error of approximation (RMSEA), which measures the difference between the hypothesized and ideal models, was observed. The value for the hypothesized model was (0.09), which is close to the acceptable value (between 0.05 and 0.08) that indicates that the model is a good fit [35]. Since the sample size was small, the comparative fit index (CFI) was utilized for verifying the model fitness, as it performs well for a small sampling. The value of CFI was 0.96, which satisfies the minimum criteria of >0.95 for a good model fit [36,37,38], as shown in Table 5 below.

#### 3.8.2. Indirect Effects of Socio-Demographics 

The survey results suggested that indirect impacts of gender, area of living, race, and education on disaster preparedness and disaster awareness are associated with the direct impacts of socio-demographics on the key variables. 

## 4. Discussion

As presented in Table 5, evaluating the relationships between socio-demographic characteristics and key variables revealed that female students are more optimistic about government agencies and universities taking responsibility for their safety during disasters than males, but they have a more negative perception of their university’s disaster curriculum. Gender is not just a factor that evaluates the distinctions between male and female in the aftermath of disasters. Additionally, it concerns how gender power relations are reflected in this situation through living situations, demographic and economic characteristics, behaviors, and attitudes [39]. Asian students and those living on campus are more positive about the curriculum and expressed willingness to take an exam that covers the material [40]. Graduate students were more aware than undergraduate students of the emergency procedures and communication channels that were established by their university.

Consideration of the mediating effects of the key variables on disaster preparedness and awareness of students showed that students who are more optimistic about the government or university assuming responsibility for their safety during a disaster and who are aware of university emergency procedures are more likely to be prepared. Those with a positive perception of DRR education in general and their university’s curriculum specifically, including being willing to take an exam at the end of the course, demonstrated a heightened awareness of disasters. 

Table 6 presents the indirect effects of sociodemographic on disaster awareness and disaster preparedness. For example, females might not be aware of disasters if they have a negative perception of the university disaster curriculum and are, therefore, unlikely to adopt DRR education. On the other hand, they are better prepared for disasters compared to males if they are optimistic about the university and government assuming responsibility for their safety during disasters. Students living on campus are likely to be less aware of disasters if they are ready to take a course on DRR education and take a test at the end of the course. Graduate students are better prepared for disasters if they are aware of university emergency procedures. Therefore, a university curriculum would help to improve disaster awareness students, which is in line with the results of previous studies [41]. 

Hence, despite the challenges of implementing DRR education as part of the curriculum, it is critical that universities provide practical training so that through practicing rescue skills, the students become more knowledgeable and proficient in how to survive a disaster. 

## 5. Conclusions

The goal of this study was to determine the DPIs and to develop models to identify the disaster preparedness of university students. The disaster preparedness indicators that were identified from the literature belong to six critical components: government/university responsibility, emergency procedures, university curriculum, adoption of disaster risk reduction, disaster preparedness, and disaster awareness. The indicators revealed that the university’s DRR curriculum significantly impacts the students’ level of disaster awareness, and the assumption of the government and university for responsibility of the students’ safety and the establishment of emergency procedures directly influence the students’ level of preparedness. The survey results indicated that the variables not only directly affect students’ disaster preparedness and awareness, but they also mediate the effects of their socio-demographic characteristics. More than half (62%) of the students who participated in the survey believed that both practical and theoretical disaster education are needed for a sound understanding of the survival techniques and rescue skills that are needed during disasters, and 31% considered lack of sufficient practical knowledge a major barrier. The findings of this study can help faculty and academic staff update existing programs and incorporate new ones. It also will allow policymakers to effectively assess the universities’ existing emergency preparedness policies and procedures based on student characteristics

As the sample size of the students that participated in the study was small and the students were only from engineering majors, the findings may not be representative of most students. In the future, more comprehensive studies should be conducted among larger groups of students from a variety of majors in disaster-prone areas in the United States to understand the factors that affect students’ disaster preparedness. Moreover, this study was developed using a self-reported questionnaire and the results are based on perceptions of disaster preparedness and not the actual disaster preparedness of students.

## Figures and Tables

**Table 1:** List of DPIs.

**#**	**Disaster Preparedness Indicators**
1	Willingness to take a DRR course
2	Confidence to assist with disaster management during emergency
3	Confidence in providing basic first aid
4	Curriculum includes psychological first- aid training
5	University has enough first-aid boxes
6	Importance of local communities to help university
7	Impact of severe natural disaster on student’s life
8	Students’ responsibility for their own safety
9	Friends’ responsibility for students’ safety
10	Parents’ responsibility for students’ safety
11	University’s responsibility for students’ safety
12	Government agencies’ responsibility for students’ safety
13	University emergency procedure awareness
14	Emergency communication system awareness during emergency
15	Curriculum includes knowledge of disaster medicine
16	Student guardian’s presence during disaster education
17	University buildings have disaster shelters
18	Importance of Local community’s role on helping university to implement DRR courses
19	Mandatory DRR education
20	Likelihood of giving a test on DRR education
21	Open to collaboration of university while handling disasters
22	University has online database regarding disaster preparedness
23	Disaster related courses taken Pre-University
24	Frequency of disaster drills practiced at university

Source: Patel et al. [26].

**Table 2:** Descriptive statistics of the survey participants.

Demographic Characteristics		#	%
Gender	Male	87	78%
	Female	24	22%
Ethnicity	African American	7	6%
	Asian	68	61%
	Hispanic	13	12%
	Other	23	21%
Levels of education	Undergraduate	39	35%
	Graduate	72	65%
Area of living	Off-Campus	78	70%
	On-Campus	33	30%
Age	Under 25 Years Old	68	61%
	Above 25 Years Old	43	39%
Annual family income	Less than $15,000	16	15%
	Less than $30,000	42	38%
	$30,000 to $60,000	17	15%
	$60,000 to $100,000	28	25%
	More than $100,000	8	7%
Involvement in number of disasters	0–1	70	63%
	1–2	13	12%
	2–3	17	15%
	3–4	3	3%
	4–5	2	2%
	More than 5	6	5%
Involvement in types of disasters (More than one response)	Tsunami	3	3%
	Hurricane	5	5%
	Tornadoes	14	13%
	Flooding	22	20%
	Thunderstorm	25	23%
	Earthquakes	28	25%
	None	50	45%

**Table 3:** Responsibility for students’ safety.

	1	2	3	4	5	6	7	Total (100%)
Myself	3 (3%)	1 (1%)	3 (3%)	4 (4%)	15 (14%)	8 (7%)	75 (69%)	109
Friends	10 (9%)	7 (6%)	14 (13%)	17 (15%)	24 (22%)	22 (20%)	16 (15%)	110
Parents	12 (11%)	10 (9%)	6 (6%)	13 (12%)	19 (18%)	15 (14%)	33 (31%)	108
University	5 (5%)	1 (1%)	6 (5%)	20 (18%)	24 (22%)	24 (22%)	30 (27%)	110
Government Agencies	4 (4%)	4 (4%)	8 (7%)	14 (13%)	15 (14%)	23 (21%)	39 (36%)	107

Rank in order of importance who you feel is responsible for your safety in the case of an emergency: 1 = “not at all important” and 7 = “extremely important”.

**Table 4:** Factor Analysis Results.

Component Name	#	Disaster Preparedness Indicators	Factor Loadings
Govt/Uni Responsibility	11	University responsibility	0.881
	12	Government agencies’ responsibility	0.894
Emergency Procedures	13	University emergency procedures	0.918
	14	Emergency communication system	0.927
University Curriculum	4	Curriculum includes first-aid training	0.683
	15	Curriculum includes disaster medicine	0.782
	16	Student guardian’s presence during disaster education	0.815
DRR Adoption	1	Willingness to take DRR course	0.791
	20	Test on DRR education	0.791
Disaster Preparedness	23	Disaster-related courses taken pre-university	0.725
	24	Frequency of disaster drills practiced at university	0.725
Disaster Awareness	22	University has online database regarding disaster preparedness	0.726
	17	University buildings have disaster shelters	−0.605
	21	Open to collaboration of university while handling disasters	0.688

**Table 5:** Direct effects given in standardized coefficients.

Critical Components		Sociodemographic	Estimate	*p*-Value
Govt/Uni Responsibility	←	Female	0.475	0.022 *
Emergency Procedures	←	Female	−0.098	0.647
University Curriculum	←	Female	−0.400	0.059 **
Govt/Uni Responsibility	←	Living on-campus	0.096	0.304
Emergency Procedures	←	Living on-campus	−0.095	0.328
University Curriculum	←	Living on-campus	0.070	0.464
DRR Adoption	←	Living on-campus	0.181	0.073 **
DRR Adoption	←	Female	−0.126	0.573
Govt/Uni Responsibility	←	Race (Asian)	0.282	0.119
Emergency Procedures	←	Race (Asian)	−0.161	0.390
University Curriculum	←	Race (Asian)	0.560	0.002 *
DRR Adoption	←	Race (Asian)	0.055	0.779
Emergency Procedures	←	Education (graduate)	0.608	0.001 *
Govt/Uni Responsibility	←	Education (graduate)	0.278	0.120
DRR Adoption	←	Education (graduate)	−0.066	0.731
University Curriculum	←	Education (graduate)	−0.026	0.886
Disaster Preparedness	←	Govt/Uni Responsibility	0.139	0.096 **
Disaster Awareness	←	University Curriculum	0.490	0.000 *
Disaster Awareness	←	DRR adoption	0.162	0.045 *
Disaster Preparedness	←	Emergency Procedures	0.178	0.027 *
Model fit	χ^2^/df < 5	RMSEA < 0.1	CFI > 0.95	
	χ^2^/df = 1.9	RMSEA = 0.09	CFI = 0.99	

Note: * significance level α = 0.05; ** significance level α = 0.10; ← = influenced by.

**Table 6:** Indirect effects on the output variables.

**Sociodemographic**	**Indirect Effects on Disaster Awareness**	**Indirect Effects on Disaster Preparedness**
Female	−0.211	0.440
Area of living (on-campus)	−0.006	−0.007
Race (Asian)	−0.338	0.022
Education (graduate)	0.060	0.062

## Data Availability

All the data that support the findings of this study are available from the corresponding author upon reasonable request.

## Supplementary Materials

The following supporting information can be downloaded at: https://www.mdpi.com/article/10.3390/ijerph20054447/s1, File S1: Disaster Preparedness Survey.
