# Supplementary material for: Disaster Preparedness and Awareness among University Students: A Structural Equation Analysis
Source: Int J Environ Res Public Health. 2023 Mar 2;20(5):4447. doi: 10.3390/ijerph20054447 (PMC10001636; doi:10.3390/ijerph20054447)
Supplement: Supplementary file 1 [file ijerph-20-04447-s001.zip › ijerph-2198082-supplementary.pdf]

Disaster Preparedness Survey

Demographic

1. Gender

    - Male
    - Female

2. Major

3. Which of the following best represent your racial or ethnic heritage?
    - Native American
    - African American
    - American Indian or Alaska Native
    - Asian
    - Hispanic
    - Native Hawaiian or Pacific Islander
    - Other

4. What is your current year of education?
    - Freshman
    - Sophomore
    - Junior
    - Senior
    - Masters
    - Ph.D.

5. How do you travel to your university?
    - Walking
    - By Personal Vehicle
    - By transit service provided by institution
    - By public transport

6. How much time does it take for you to reach to your university?
    - Less than 10 Minutes
    - 10 - 20 Minutes
    - 20 - 30 Minutes
    - 30 - 60 Minutes
    - More than an hour

7. Do you live off-campus or on-campus housing?
    - Off - Campus
    - On – Campus

8. Please identify your annual family income.
    - Less than 15,000
    - 15,000 to 30,000
    - 30,000 to 60,000
    - 60,000 to 100,000
    - More than 100,000

Disaster Experience

9. What type of disasters have you been involved in? If yes, please check all that apply.

    Hurricane
    Tornadoes
    Flooding
    Thunderstorms
    Earthquakes
    Tsunami
    None

10. Approximately how many disasters have you been involved in?
    - 0 - 1
    - 1 - 2
    - 2 - 3
    - 3 - 4
    - 4 - 5
    - More than 5

11. What was the maximum loss you suffered due to a disaster?

- Loss of a family member
- Loss of a friend
- Suffered physically
- Suffered mentally
- Did not suffer
- Don’t want to answer

Disaster Risk Reduction Education

Disaster risk reduction (DRR) is a systematic approach to identifying, assessing and reducing the risks of disaster. It aims to reduce socio-economic vulnerabilities to disaster as well as dealing with the environmental and other hazards that trigger them.

12. How important it is to include DRR education in your curriculum?

	Not at all Important	Slightly Important	Somewhat Important	Moderately Important	Very Important	Quite Important	Extremely Important
Importance of DRR being included in Curriculum	❑	❑	❑	❑	❑	❑	❑

13. Have you taken any DRR courses in the past? If yes, please specify at what level of education were you undergoing while taking DRR course.
    - Elementary School
    - Middle School
    - High School
    - Undergraduate
    - Graduate
    - Doctoral
    - Other

14. How many disasters related courses were you taught during your pre-university education?
    - 0 - 1
    - 1 - 2
    - 2 – 3
    - 3 – 4
    - 4 - 5
    - More than 5

15. How willing are you to take a Disaster Risk Reduction course?

	Not at all Willing	Slightly Willing	Somewhat Willing	Moderately Willing	Very Willing	Quite Willing	Extremely Willing
Willingness to take DRR course	❏	❏	❏	❏	❏	❏	❏

16. What form of DRR education do you think needs to be incorporated in your curriculum?
- o Practical
- o Theory
- o Both Practical and Theory
- o None

17. How often should DRR education be incorporated in your curriculum?
- o Every semester
- o Once a year
- o Once every two years
- o Once throughout the entire course
- o None

18. Do you agree that having proper knowledge about the disaster would help you handle the loss due to the disaster?
- o Extremely helpful
- o Moderately helpful
- o Slightly helpful
- o Not helpful

Emergency Awareness

19. How confident are you to assist with disaster management during emergency?

	Not at all Confident	Slightly Confident	Somewhat Confident	Moderately Confident	Very Confident	Quite Confident	Extremely Confident
Confidence to assist with disaster management during	❏	❏	❏	❏	❏	❏	❏

emergency situation							

20. How confident are you in providing basic first aid in emergency situations?

	Quite Confident	Extremely Confident	Not at all Confident	Slightly Confident	Somewhat Confident	Moderately Confident	Very Confident
Confidence in providing basic first aid	❑	❑	❑	❑	❑	❑	❑

21. Do you have proper knowledge regarding what kind of wound can happen due to a disaster and how it can be handled primarily through first-hand aid?
    - Yes, during disaster drill, my school teaches us that.
    - Yes, I learned it from outside.
    - No, my school does not have such training.

22. Is your curriculum providing psychological first aid training?

	Definitely yes	Probably yes	Might or might not	Probably not	Definitely not
Curriculum Provides Psychological First Aid Training	❑	❑	❑	❑	❑

23. Does your university have enough first aid boxes and tools?

	Probably not	Definitely not	Definitely yes	Probably yes	Might or might not
Enough First Aid Boxes and Tools	❑	❑	❑	❑	❑

24. How important is it for the local communities to help universities during emergency situations?

	Not at all Important	Slightly Important	Somewhat Important	Moderately Important	Very Important	Quite Important	Extremely Important
Importance of local communities to help university	❑	❑	❑	❑	❑	❑	❑

25. If a severe natural disaster happens in the university, how impactful could it be in your life as a student?

	Not at all Impactful	Slightly Impactful	Somewhat Impactful	Moderately Impactful	Very Impactful	Quite Impactful	Extremely Impactful
Impact of Natural Disaster	❑	❑	❑	❑	❑	❑	❑

26. Does your university have a nursing department? If yes, does having a nursing department makes you confident regarding your university's preparedness of disaster?
    - o Yes it does
    - o No it does not
    - o My university does not have nursing department

27. In the case of emergency who is responsible for your safety, rank in order of importance.

	Not at all Important	Slightly Important	Somewhat Important	Moderately Important	Very Important	Quite Important	Extremely Important
Myself	❑	❑	❑	❑	❑	❑	❑
Friends	❑	❑	❑	❑	❑	❑	❑
Parents	❑	❑	❑	❑	❑	❑	❑
University	❑	❑	❑	❑	❑	❑	❑
Government Agencies	❑	❑	❑	❑	❑	❑	❑

28. Do you think that the disaster would harm you less if you were at home when it attacked?
    - o Yes, my home is safer.
    - o Yes, my home is outside of the disaster area.
    - o No, my school is safer
    - o No, it does not make any difference
    - o I don't know

Emergency Drills in University

29. How aware are you of the emergency procedures in your university?

	Extremely Unaware	Moderately Unaware	Slightly Unaware	Somewhat Aware	Slightly Aware	Moderately Aware	Extremely Aware
Awareness for Emergency Procedures in Your University	❑	❑	❑	❑	❑	❑	❑

30. How well are you aware of the modes of communication system provided by your university during emergency?

	Extremely Unaware	Moderately Unaware	Slightly Unaware	Somewhat Aware	Slightly Aware	Moderately Aware	Extremely Aware
Awareness of communication system provided by your university	❑	❑	❑	❑	❑	❑	❑

31. Does your university have an online database regarding disaster preparedness?

	Definitely not	Definitely yes	Probably yes	Might or might not	Probably not
University has online database regarding disaster preparedness	❑	❑	❑	❑	❑

32. Was your university able to provide bulletin regarding an upcoming disaster? If yes, what was the medium of prediction?
   - Email
   - Class Announcement
   - Did nothing, I came to know from other sources

33. How often are disaster drills are practiced in your university?
   - Every semester
   - Once a year
   - Once every two years

- Once throughout the entire course
- Never

34. Does the DRR curriculum or disaster drill include knowledge regarding disaster medicine?

	Definitely yes	Probably yes	Might or might not	Probably not	Definitely not
Curriculum or disaster drill include knowledge regarding disaster medicine	☐	☐	☐	☐	☐

35. Does the building where you attend classes have a disaster shelter?

	Probably yes	Might or might not	Probably not	Definitely not	Definitely yes
Building where you attend class have a disaster shelter	☐	☐	☐	☐	☐

36. Does your university include your guardian while providing disaster preparedness education?

	Definitely yes	Probably yes	Might or might not	Probably not	Definitely not
University include guardian while providing disaster preparedness education	☐	☐	☐	☐	☐

37. Are you open to collaboration when it comes to handling a disaster?

	Definitely yes	Probably yes	Might or might not	Probably not	Definitely not
Open to collaborate while handling a disaster	☐	☐	☐	☐	☐

Implementation of DRR Courses

38. How important it is for the local entities to help universities to implement DRR courses?

	Quite Important	Extremely Important	Not at all Important	Slightly Important	Somewhat Important	Moderately Important	Very Important

Importance of Local entities helping university to implement DRR Courses	❑	❑	❑	❑	❑	❑	❑

39. Does your university team up with experts at DRR forums to invite them to provide credible information to the students? If yes, what is the frequency?
    - They never came
    - They came once
    - They come once a year
    - They come once a semester
    - I don't know

40. According to you what are the major barriers to learn DRR education?(Can mark multiple options)
    - Not enough exposure to practical knowledge.
    - Lack of previous exposure to disasters.
    - Not enough disaster preparedness drills.
    - Lack of direct engagement with local disaster management agency.
    - Lack of knowledge in developing curriculum for disaster Education.
    - Lack of trained staff in the university.
    - Other please specify

41. Is taking course related to DRR preparedness mandatory in your university?
    - Yes, it is mandatory
    - No, it is not mandatory
    - My university does not offer any courses

42. Do you agree that the education system should make DRR education mandatory?

	Neither Agree nor Disagree	Somewhat Agree	Agree	Strongly Agree	Strongly Disagree	Disagree	Somewhat Disagree
DRR education should be made mandatory by education system	❑	❑	❑	❑	❑	❑	❑

43. Whom do you feel comfortable learning DRR education from?
    - Professors
    - University Staff
    - Experienced Private Sector Professionals (With more than 10 years of experience)
    - Experienced Government Sector Professionals (With more than 10 years of experience)

o All of the above

44. How likely would you give a test based on DRR education if it is in your curriculum.

	Moderately unlikely	Slightly unlikely	Neither likely nor unlikely	Slightly likely	Moderately likely	Extremely likely	Extremely unlikely
Likelihood of giving test based on DRR education	❑	❑	❑	❑	❑	❑	❑
